# The alternative androgen receptor isoform A mitigates toxicity of polyglutamine-elongated mutant androgen receptor in spinal and bulbar muscular atrophy

**DOI:** 10.1016/j.jare.2026.01.051

**Published:** 2026-01-19

**Authors:** Marta Chierichetti, Roberta Andreotti, Barbara Tedesco, Veronica Ferrari, Laura Cornaggia, Paola Pramaggiore, Marta Cozzi, Ali Mohamed, Rocio Magdalena, Margherita Piccolella, Giulia Boarolo, Valeria Crippa, Paola Rusmini, Mariarita Galbiati, Carlo Rinaldi, Eric N. Anderson, Udai Bhan Pandey, Maria Pennuto, Riccardo Cristofani, Angelo Poletti

**Affiliations:** a Dipartimento di Scienze Farmacologiche e Biomolecolari “Rodolfo Paoletti”, Dipartimento di Eccellenza 2018-2027, Università degli Studi di Milano, Milan, Italy; b Department of Biomedical Sciences, University of Padova, Padova, Italy; c Veneto Institute of Molecular Medicine (VIMM), Padova, Italy; d Department of Pediatrics, University of Oxford, Oxford, UK; e Department of Pediatrics, Children’s Hospital of Pittsburgh, University of Pittsburgh Medical Center, Pittsburgh, PA, USA

**Keywords:** Androgen receptor, Polyglutamine tract, CAG repeat, Motoneuron diseases, Neuromuscular diseases, spinal and bulbar muscular atrophy (SBMA), Kennedy’s disease

## Abstract

**Introduction:**

Spinal and bulbar muscular atrophy (SBMA) is an X-linked neuromuscular disease caused by a CAG-repeat expansion in the androgen receptor (AR) gene, translated into an elongated polyglutamine (polyQ) tract in the protein. Androgens trigger ARpolyQ toxicity, thus most potential therapeutic approaches involve androgen reduction or AR negative modulation, with severe endocrine side effects.

**Objective:**

A start codon (I-AUG) controls AR translation, while a second one, (II-AUG) downstream to I-AUG and the CAG repeat, drives translation of the shorter AR isoform (AR-A) lacking the polyQ tract, but preserves all relevant AR functional domains. Here, we studied AR-A behaviour and its effect on ARpolyQ toxicity in SBMA.

**Methods:**

We took advantage of cellular, mice and *Drosophila melanogaster* SBMA models to asses AR-A behaviour and effect on ARpolyQ aggregation and functions.

**Results:**

Comparative expression analyses of AR isoforms revealed that AR-A is predominantly located in the mouse brainstem and spinal cord of the central nervous system, but not in muscle. AR-A retains partial androgenic activity, but does not aggregate. Since ARpolyQ and AR-A can heterodimerize, we tested the effect of AR-A on ARpolyQ behaviour, showing that AR-A has a pro-solubilizing effect on ARpolyQ aggregates and that the heterodimers retain a partial transcriptional activity. Finally, we tested the effect of AR-A in a fly model of SBMA. Flies expressing AR-A alone showed no signs of external eye degeneration, in contrast to those expressing expanded ARpolyQ. Notably, co-expression of AR-A with ARpolyQ significantly reduced ARpolyQ aggregation and eye degeneration, supporting our hypothesis that AR-A enhances ARpolyQ solubility and mitigates its toxicity *in vivo*.

**Conclusions:**

Altogether, our results demonstrate that an increased expression of AR-A may have a role in protecting against ARpolyQ aggregation and toxicity. These findings suggest that AR-A could represent a promising avenue for developing an alternative therapeutic strategy that warrants further investigation.

## Highlights

• Here we characterized the AR-A expression in adult murine tissues, finding its presence in the spinal cord and brainstem.
• We found AR-A does not aggregate and translocate into the nuclei, activating AR-dependent transcription.
• AR-A exerts a pro-solubilizing effect on different ARpolyQ aggregates, reducing their conversion into insoluble species.
• In vivo evidence in flies showed that AR-A suppresses ARpolyQ-induced eye neurodegeneration.
• Collectively, our data suggest that AR-A induction may be a potential therapeutic intervention for SBMA.

## Graphical abstract

**Figure:**
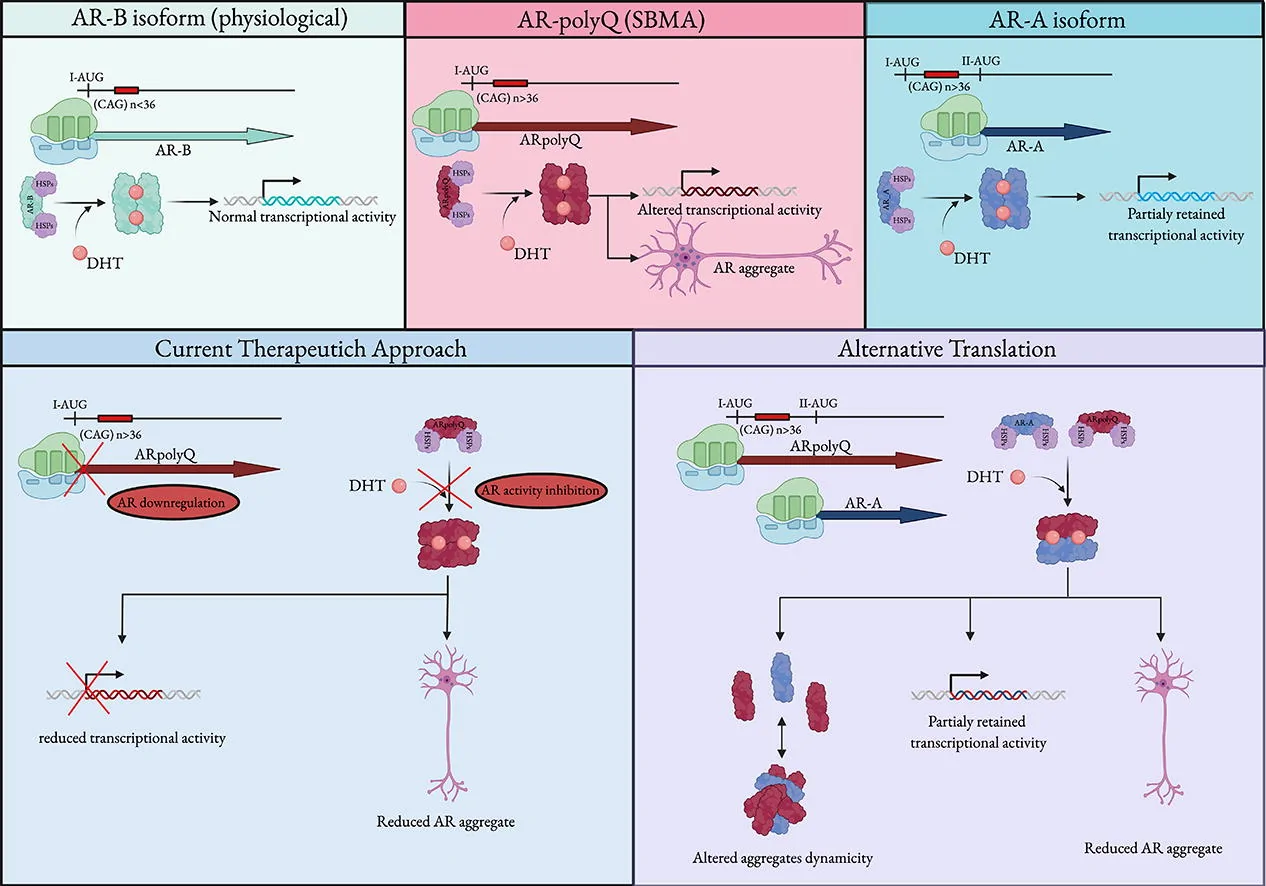

## Introduction

Spinal and bulbar muscular atrophy (SBMA), also known as Kennedy’s disease, is an X-linked hereditary and slowly progressive neuromuscular disease (NMD) affecting only males and typically manifests between the third and fifth decade of life [[1], [2], [3]]. The underlying cause is a trinucleotide (CAG) repeat expansion in the first exon of the androgen receptor (*AR*) gene, which is translated into a polyglutamine (polyQ) tract in the AR protein [1,4,5], thus belonging to the CAG/polyglutamine-related diseases [6]. The CAG repeat is polymorphic in length, ranging between 9 and 36 repeats in the normal population, while in patients it becomes longer than 37 contiguous CAGs with a maximum size of 72 repeats reported so far [7]. The length of the CAG expansion partially correlates with the age of onset [8]. The disease is relatively rare, with founder effects in specific geographic areas. The global prevalence is approximately 2.58/100,000 males [9] though it is probably largely underestimated, since a four-fold prevalence of subjects carrying pathological CAG repeats in the *AR* gene has been reported in the global population [10], most likely with no SBMA symptoms. Thus, the number of individuals at risk of developing SBMA in their lifetime is likely underestimated [11]. In addition, SBMA is still frequently misdiagnosed with other NMDs, or underdiagnosed for reduced penetrance and/or pleomorphic clinical manifestations.

In general, initial symptoms include proximal lower limb weakness, muscle cramps, and tremors, followed by sensory loss accompanied by facial, bulbar, and limb muscular weakness and atrophy, associated with endocrinological abnormalities [12]. The neuromuscular symptoms are linked to the loss of lower motor neurons in the brainstem and in the anterior horns of the spinal cord, and to muscle damage [13,14]. However, the relationship between these two ARpolyQ-targeted structures is complex and not fully understood. In fact, muscle degeneration or motoneuronal loss are both consequences of the loss of the neuromuscular dialog with neuromuscular junction impairment, chronic denervation, and direct effects of ARpolyQ toxicity on motoneuronal cells and muscle fibers [[14], [15], [16], [17]]. SBMA pathogenesis is related to the presence of testosterone or its metabolite dihydrotestosterone (DHT). In fact, unbound AR is retained in the cytoplasm interacting with different chaperone proteins (HSP90, HSP40, and HSP70), that maintain the ligand-binding pocket in a high-affinity conformation for the ligand [18] and possibly mask the polyQ. Upon testosterone binding, AR dissociates from the accessory HSPs, dimerizes, undergoes conformational changes that expose the nuclear localization signal (NLS), and translocates into the nucleus to regulate transcription [19,20]. The expanded polyQ tract in the mutant AR alters several of these processes, triggering ARpolyQ toxicity, possibly by exposing the polyQ and acquiring aberrant conformations [19,21]. This results in ARpolyQ aggregation in both neuronal and non-neuronal tissues [22], representig a typical toxic gain of function (GOF) mechanism strictly linked to the polyQ presence and implying a number of other downstream alterations, including the depletion of components of the protein quality control (PQC) system [[22], [23], [24]] and transcriptional alterations [25]. Thus, ARpolyQ aggregation is a crucial event that triggers and contributes to the degeneration of neuronal and muscle cells, which are highly vulnerable to proteotoxicity due to the low or absent turnover of proteins.

Multiple *AR* isoforms exist, which might arise from alternative splicing events, generating alternative transcripts, or alternative usage of different start codons. Intriguingly, multiple alternative AR transcripts have been identified in patients induced pluripotent stem cells (IPSCs)-derived motor neurons. These include the AR45, a 45 kDa isoform that has been identified as a negative modulator of AR transcriptional activity, which restores the transcriptional profile and the pathological degeneration in SBMA models without altering inclusion accumulation [26]. Instead, different translation products are generated through the alternative use of different start codons downstream to the canonical one (I-AUG). This is the case of AR-A, an isoform with an apparent molecular mass of 87 kDa. The AUG starting codon for the full-length (FL)-AR (I-AUG) is located 1130 bases downstream of the transcription start site (TSS) and drives the translation of either the wild type (WT) AR (also named AR-B) or the mutated ARpolyQ (in the normal population or in SBMA patients, respectively) (**1A**). The II-AUG codon is located downstream the CAG repeat encoding the polyQ tract, leading to the translation of AR-A, which lacks the first 188 amino acids (aa) of the N-terminal domain (NTD) including the polyQ tract [27], thus being potentially not toxic to cells. The role of the AR-A isoform in both physiological and pathological conditions has been poorly investigated and remains virtually unknown. Despite AR-A was reported in 1994 by Wilson and colleagues within human genital skin fibroblast cells expressing a mutated AR (R776) [27], few studies focused on its expression [[28], [29], [30], [31]]. This gap of knowledge is mainly due to the great difficulties in discriminating between the expression of the two isoforms with the currently available antibodies recognizing the AR proteins. Indeed, the AR-B isoform is detected by antibodies that recognize the NTD, an internal sequence within the NTD, or the C-terminal domain. In contrast, AR-A is recognized by anti-AR antibodies directed against AA 200–220 or the C-terminal domain, but not by the antibodies that recognize AA 1–21. The AR-B is the predominant isoform, with an AR-B:AR-A ratio of approximately 10:1 in androgen-responsive tissues. Interestingly, this ratio remains relatively consistent across different fetal and adult tissues. AR-A generally constitutes less than 20% of the total AR in reproductive tissues and less than 26% in any tissue [28]. However, data on AR-A abundance in adult neuronal and muscular tissues, which are mostly involved in SBMA, are currently lacking. Despite this, the fact that AR-A is devoid of the toxic polyQ tract makes this isoform very attractive for therapeutic purposes. In fact, no approved therapies exist to prevent or halt SBMA progression and patients rely on limited palliative treatments to manage symptoms. The main potential therapeutic approaches attempted so far are based on the suppression of AR expression as well as on blockage of androgen synthesis or functions to prevent the androgen-induced toxic properties of ARpolyQ, but they are linked to severe endocrine side effects for SBMA patients [32]. Therefore, identifying novel therapeutic strategies targeting aggregate reduction without compromising AR function is crucial.

To pursue this strategy, it is relevant to ascertain whether the lack of the first 188 aa may impair or affect AR-A functions. In fact, upon binding to androgens, AR-B is activated, hyperphosphorylated, and dimerizes translocating into the nucleus, where it binds androgen-responsive elements (AREs) to regulate transcription in tissue- and cell-specific manners. The physiological polyQ tract apparently represses AR-B transcriptional activity, while the pathological elongated polyQ exacerbates this repressive function [2,33]. This could be explained by the formation of an aberrant helical structure stabilized by unconventional hydrogen bonds between glutamine side chains and main chain carbonyl groups, that directly correlate with the length of the polyQ tract. The aberrant hydrogen bonds are bifurcate with the conventional hydrogen bonds stabilizing α-helices [34]. In addition, adjacent to the polyQ tract resides a FQNLF motif (attributable to the canonic FxxLF recognition motif), involved in AR phosphorylation (e.g.: Ser 215) and Steroid receptor coactivator-3 binding, allowing transcription modulation [35]. This FQNLF motif is also involved in HSP70 binding [36]. The same motif interacts with the AR ligand-binding domain (LBD), generating the AR N/C interaction upon androgen activation; thus, molecular chaperones and the LBD compete for binding to the FQNLF motif, modulating AR activation. These factors control both AR stability and activation and are disturbed by the elongated polyQ tract. Indeed, a partial AR loss of function (LOF) contributes to some aspects of SBMA (i.e. androgen insensitivity), but motor neurons (MN) loss and muscle atrophy are largely due to the AR GOF triggered by androgens via misfolding, aggregation, and/or formation of toxic oligomeric species. All these dysfunctions should not be present in AR-A, which lacks the relevant regions for these activities.

Based on these observations, in this study, we have characterized the expression of the AR-A isoform in SBMA-affected tissues and evaluated its biochemical properties, both when expressed alone or in combination with the mutant ARpolyQ. Our data indicate that AR-A is expressed in the brain and spinal cord of mice and its over-expression reduces the toxicity of ARpolyQ in motoneuronal cells. This leads to the hypothesis that the induction of the translation from the II-AUG or the AR-A delivery in affected cells may protect against SBMA pathology.

## Materials and methods

### Chemicals

The subsequent reagents were purchased by Merck-Millipore (Burlington, MA, USA) 5α-Dihydrotestosterone (DHT, A8380), Z-Leu-Leu-Leu-al (MG132, C2211). 3‐ Methyladenine was purchased by Selleck Chemicals (3MA, S2767, Selleckchem, Huston, TX, USA).

### Cell Cultures

Murine Neuroblastoma x Spinal Cord 34 (NSC-34) cells were kindly provided by Dr. N.L. Cashman (University of British Columbia, Vancouver, BC, Canada) and are routinely use in our laboratory [[37], [38], [39], [40]]. Cells were grown in Dulbecco’s modified Eagle’s medium (DMEM) (ECB7501L, Euroclone) supplemented with Penicillin-Streptomycin solution 100X (ECB3001D, Euroclone), 1 mM L-glutamine (ECB3004D, Euroclone), and 5 % charcoaled fetal bovine serum (FBS) (F7524, Sigma-Aldrich).

Human embryonic kidney (HEK293T, ATCC) cells were cultured and plated in DMEM [supplemented with 10 % fetal bovine serum (FBS, Life Technologies, #10270106), 1 % penicillin/streptomycin (pen/strep, ECB3001D, Euroclone ), and 1 % L-glutamine]. Cells were cultured in a humidified incubator with 5 % CO_2_ at 37 °C.

### Transfection and treatments

pEGFPN1 plasmid (U55762, Clontech Lab) was used to evaluate transfection efficacy in experiments performed with untagged plasmids. pcDNA3.1 (V790-20, Invitrogen) was used as empty vector control and to normalize the total amount of transfected DNA. pGRE2E1bLuc is a luciferase reporter plasmid driven by two AREs from the tyrosine amino transferase promoter, followed by the adenovirus E1b TATA box [41], kindly obtained from Dr. N.L. Weigel (Baylor College of Medicine, Houston, TX, USA). pCMV-βgal plasmid, encoding β-galactosidase (6177–1, Clontech), was used to normalize transfection efficiency in transcriptional activity assay. pEGFP-ARQ22 plasmid coding for the AR containing 22 glutamines was obtained as previously described [42]. pEGFP-ARQ66 plasmid coding for the AR containing 66 glutamines was obtained from pcDNA5/TO-GFPARQ66 (kindly provided by Prof. Heather L. Montie (Philadelphia College of Osteopathic Medicine, Philadelphia, PA, USA) cloning the HindIII GFP-ARQ66 fragment into pEGFP-ARQ0 plasmid [42]. pARQ22 and pARQ66 were obtained by excision of the GFP sequence from the corresponding GFP-AR plasmids. pEGFP-AR-A and pAR-A were obtained by excision of the I-ATG site respectively from pEGFP-ARQ22 and pARQ22 through SalI and AflII restriction enzymes. RFP-AR-A was obtained starting from the pEGFP-AR-A plasmid by substituting the GFP sequence with the RFP sequence (derived from pRFP-LC3 (200943, Addgene) through BglII and AflII restriction enzymes. NSC-34 cells were transfected 24 h after seeding with Lipofectamine® 3000 Transfection Reagent (L3000015, Invitrogen, Life Technologies Corporation, Carlsbad, CA, USA). Cells were transfected following the manufacturer's protocol with the following amounts of reagents: i) for western blot and Filter Retardation assay analyses, 1 μg of total plasmid DNA (0.5 μg of pARs and 0.5 pCDNA3.1), 2 μL of Lipofectamine® 3000, and 2 μL of P3000 Reagent. ii) For immunofluorescence analysis, 0.6 μg of total plasmid DNA (0.3 μg of pGFP-ARs or pRFP-AR and 0.3 μg of pcDNA3.1 plasmid), 1 μL of Lipofectamine® 3000, and 1 μL of P3000 Reagent. iii) For transcriptional activity assay, 0.2 μg of pARs, 0.2 μg of pGRE2E1bLuc, 0.1 μg of pCMV-β, 0.05 μg of pEGFPN1, 0.25 μL of Lipofectamine® 3000, and 0.25 μL of P3000 Reagent. iv) For FloIT, 0.5 μg of pGFP-ARs or pAR, 1 μL of Lipofectamine® 3000, and 1 μL of P3000 Reagent. For live imaging analysis, 1.5 μg of pARs, 2 μL of Lipofectamine® 3000, and 2 μL of P3000 Reagent. HEK293T cells were transfected with polyethyleneimine (PEI, branched MW 25,000 Da, 408727, Sigma-Aldrich) according to well dimensions. DNA:PEI (0.5 %v/v) ratio was 1:1. At 24 h after the transfection the DMEM complete medium supplemented with 10 % FBS was changed to DMEM supplemented with Charcoal Stripped FBS (CS-FBS) and cells were treated with vehicle (ethanol) or 10 nM DHT and harvested for the different analyses 24 h post-treatment.

### Western blot and filter retardation assay

For Western blot (WB) analysis and Filter Retardation Assay (FRA), NSC-34 cells were plated in a 12 multiwell plate at 80.000 cells/well. After 24 h cells were transfected and treated with EtOH or 10 nM DHT. 48 h after transfection, cells were harvested and centrifuged for 5 min at 100 g at 4 °C. Pellet was resuspended in PBS (P4417, Sigma-Aldrich) added with protease inhibitor (P8340, Sigma-Aldrich), and homogenized using slight sonication (3 cycles at 10 % intensity). For WB, 15 μg of total proteins were added with sample buffer (0.6 % Tris, 2 % SDS [L3771, Sigma-Aldrich], 10 % glycerol [G5516, Sigma-Aldrich], 5 % β-mercaptoethanol [M3148, Sigma-Aldrich,], pH 6.8) and heated to 100 °C for 5 min. Samples were loaded onto 7.5 % SDS polyacrylamide gels and transferred to nitrocellulose membranes (10600003, Amersham™ Protran™ Premium 0.45 μm NC) by Trans-turbo Transfer System (Mini Trans-Blot Cell; Bio-Rad Laboratories). Membranes were then incubated for 1 h at RT with a blocking solution of 5 % dried non-fat milk (NB0669, BioBasic) in TBS-T (Tris base 20 mM, NaCl 140 mM, 0.05% Tween-20; pH 7.6) and then with the following primary antibodies diluted in blocking solution overnight at 4 °C: i) rabbit monoclonal anti-AR (dilution 1:3,000 ab52615, Abcam Inc.) to detect all the AR isoforms. ii) rabbit polyclonal anti-GAPDH (dilution 1:5,000, sc-25778, Santa Cruz Biotechnology) to detect GAPDH, used as a loading control. Immunoreactivity detection was performed using goat anti-rabbit peroxidase-conjugated secondary antibodies (dilution 1:10,000, 111–035-003 and 115–035-003, Jackson ImmunoResearch) and Westar Antares ECL detection reagent (XLS142, Westar Antares, Cyanagen). Image acquisition was performed using a ChemiDoc XRS System (Bio-Rad, Laboratories). The optical density of the bands was analyzed using Image Lab Software (Bio-Rad Laboratories).

For FRA, 6 μg of total proteins were analyzed using a Bio-Dot SF Microfiltration Apparatus (Bio-Rad) and a 0.2 μm cellulose acetate membrane (100404180, Amersham). Membranes were processed as previously described for WB with the following primary antibody: i) Rabbit Monoclonal anti-AR (dilution 1:3,000, ab133273, Abcam Inc.) to specifically detect only AR-B or ARpolyQ. Immunoreactivity detection was performed using a goat anti-rabbit peroxidase-conjugated secondary antibody (dilution 1:5,000, 111–035–003, Jackson ImmunoResearch) and Westar Antares ECL detection reagent (XLS142, Westar Antares, Cyanagen). Image acquisition was performed using a ChemiDoc XRS System. Optical density of the bands was analyzed using Image Lab Software.

HEK293T cells were harvested and lysed for WB analysis in RIPA buffer (6 mM Na_2_HPO_4_, 150 mM NaCl, 4 mM NaH_2_PO_4_, 2 mM EDTA pH 8.0, 1 % sodium deoxycholate, 0.5 % Triton X-100) plus fresh protease inhibitors. Samples were incubated for 20 min on ice and then centrifuged for 15 min at 21,000 *g* at 4 °C. Supernatant was collected and stored at − 80 °C or further processed for WB. Membranes were incubated with anti-AR (abcam 52615) and anti-tubulin (T7816, Sigma).

For *in vitro* dephosphorylation assay, HEK cells were lysed in a specific lysis buffer (50 mM Tris pH 7.5, 1 % Triton, and protease inhibitors), sonicated and centrifugated at 4 °C at 21,000 *g*. They were then incubated with 1500 Units of l phosphatase (l PP, NEB P0753S) for 6 h at 30 °C in 1X NEB Buffer Pack for Protein Metallo Phosphatases (PMP, NEB B0761S) supplemented with 1 mM MnCl_2_ (NEB, B1761). Samples were then processed for WB analysis.

### Protein extraction and analysis from mouse tissues

Frozen tissues were pulverized using a mortar and pestle on dry ice, transferred to a cold sample tube, and resuspended in 2 % SDS-RIPA buffer (50 mM Tris–HCl pH 8.0, 150 mM NaCl, 1 % NP40, 0.5 % sodium deoxycholate, 2 % SDS) with fresh protease inhibitors (P8340, Sigma-Aldrich,). Lysates were sonicated and centrifuged at 21,000 *g* for 15 min at 4 °C. Protein concentration was measured using bicinchoninic acid assay (Pierce BCA Protein Assay, Thermo Fisher Scientific). For WB, equal amounts of protein extracts from tissues were boiled at 95 °C for 5 min in 5X sample buffer (62.5 mM Tris–HCl pH 6.8, 2 % SDS, 25 % glycerol, 0.05 % bromophenol blue, 5 % β-mercaptoethanol) and separated by SDS-PAGE. Proteins were transferred to 0.45 μm nitrocellulose membranes (162-0115, Bio-Rad), blocked for 1 h in 5 % non-fat milk in TBS buffer/0.1 % Tween, and incubated with primary antibodies (anti-AR, Abcam 52615, 1:1,000 in 5 % non-fat milk TBS-T) overnight at 4 °C. HRP-conjugated secondary antibodies were incubated for 1 h at RT (1:5,000 dilution in blocking solution, Bio-Rad, goat anti-rabbit 1706515), and signals were detected with Alliance Q9 Mini Chemidoc system (Uvitec, Cambridge, UK).

### Fluorescence microscopy analysis

NSC-34 cells were seeded at 25,000 cells/well on 13 mm-diameter coverslips in MW24 plates, transfected, and treated with EtOH or 10 nM DHT. 48 hours after transfection, cells were fixed with a 4 % paraformaldehyde in 0.2 M PB solution (KH_2_PO_4_ (0.06 M) and Na_2_HPO_4_ (0.26 M)) and stained with 4′,6-diamidino-2-phenylindole (DAPI) 1:10,000 in PBS (D9542, Sigma) to visualize the nuclei. Coverslips were mounted using Mowiol® 4–88 (475,904, Merck-Millipore, Burlington, MA, USA), acquired with a Zeiss LSM900 laser scan microscope, and analyzed with Zen software (Ver. 3.7; Zeiss).

### Live imaging and FRAP

NSC-34 cells were seeded at 5000 cells/well in a 35 mm petri dish with 10 mm-diameter coverslips (P35G-0–10-C, MatTek), transfected, and treated with EtOH or 10 nM DHT. 48 h after transfection cells were analyzed with a Zeiss LSM900 laser scan microscope. Images were acquired every 2 s for 154 s. After 4 s of acquisition, the selected fields were photobleached with 100 iterations. Images were analyzed with Zen software (Ver. 3.7; Zeiss).

### Transcriptional activity

NSC-34 cells were plated at 5000 cells/well in white MW96 plates, transfected, and treated with EtOH or 10 nM DHT. 48 h after transfection cells were lysed with 40 μl/well of a 1:1 solution of white DMED (ECB7504, Euroclone) and SteadyLite Plus (6066751, Perkin Elmer) avoiding direct light. Samples were incubated for 10 min at RT and luminescence was analyzed using Enspire plate spectrofluorometer (PerkinElmer). Samples were subsequently added with 80 μl/well of assay buffer (60 mM Na_2_HPO_4_ 40 mM NaH_2_PO_4_ 10 mM KCl, 1 mM MgSO_4_, 50 mM β-mercaptoethanol, pH 7.0) and 25 μl/well of β-galactosidase substrate O-nitrophenyl-β-D-galactopyraniside (ONPG, 4 mg/ml in water; N1127, Sigma-Aldrich). After incubation at 37 °C until yellow colour appeared, 420 nm absorbance was evaluated using Enspire plate spectrofluorometer (PerkinElmer).

### FloIT

NSC-34 cells were plated at 50,000 cells/well in MW24 plates, transfected, and treated with EtOH or 10 nM DHT. Cells were harvested and centrifuged at 100 *g* for 5 min and resuspended in 500 ul PBS w/o Ca^2^^+^ and Mg^2^^+^ added with 2.5  mM EDTA and 5 % of FBS. Transfection efficacy of alive cells was analyzed using 150  μl of cell suspension added with DRAQ7 dye (D15106, ThermoFisher), used to exclude non-viable cells, and a non-transfected sample was used to set a GFP-positive threshold. Photomultiplier (PMT) voltages of 540 V for DRAQ7 and 373 V for GFP were used, and a Forward Scatter (FSC) threshold of 100,000 was set to exclude debris. The aggregate number was evaluated using 150 μl of cell suspension added with 150 μl of lysis buffer [PBS w/o Ca^2^^+^ and Mg^2^^+^ added with 1 % (v/v) Triton X-100 and protease inhibitor cocktail (Roche)] and DAPI (1:50,000) to identify nuclei. Additionally, to set the DAPI auto-fluorescence gate, one control sample was added with lysis buffer without DAPI. Samples were incubated for 2 min at RT and then analyzed using FSC and side scatter, DAPI (501 V PMT), and GFP (482 V PMT) fluorescence. A 1000 threshold (corresponding to standards microspheres diameter smaller than 2  μm as indicated in the NovoCyte® Flow Cytometer Operator's Guide) was used to minimize exclusion of small inclusions and all axes were set to log10. DAPI fluorescence and FSC-A parameters were used to identify nuclei while GFP fluorescence and FSC-H parameters were used to quantify inclusions in the non-nuclear events population. FloIT analysis was performed with a Novocyte 3000 flow cytometer (ACEA biosciences) using the subsequent setup for fluorescence analysis: a) 640  nm excitation, 660/20  nm emission for DRAQ7, b) 405  nm excitation, 445/45  nm emission for DAPI and c) 488  nm excitation, 530/30  nm emission for GFP. Data were analyzed with NovoExpress software (version 1.4.1; ACEA biosciences) and the number of inclusions was calculated as described by Whiten and colleagues [43].

### Animal models

Animal care protocols follow the appropriate national legislation and guidelines of the Council of the European Communities. This study was conducted in accordance with institutional guidelines and was approved by the Ethics Committee of the University of Padua and the Italian Ministry of Health (approval number 207/2020-PR). The mice were pathogen-free according to the FELASA list (FELASA 2014). Animals were housed in a single ventilated cage with autoclaved commercial soil bedding, food, and enrichment. Mice were housed in filtered cages in a temperature-controlled room with a 12-hour light/dark cycle with ad libitum access to water and food. We used wild type (WT) and knock-in SBMA (KI) mice in which AR exon 1 was replaced with the human AR exon 1 coding for a polyQ AR with 24Q for the WT and 113Q for the KI mice. Mice were genotyped by PCR on ear punch DNA using RED Extract-N-Amp Tissue PCRn kit according to manufacturer’s instructions, using the following primers: AR113Q Forward 5′CCACGTTGTCCCTGCTGGGCCCCAC; Reverse 5′ GACACTGCTTTACACAACTCCTTGGC.

The PCR product was run on 3 % Agarose gel for AR113Q mice in Tris-Acetate-EDTA (TAE) 1X Buffer at 80 V for 30 min [21].

The research done in flies was approved by the University of Pittsburgh Institutional biosafety office. All *Drosophila* stocks were maintained on standard cornmeal medium and fed with 2 mM DHT at 28 °C in light/dark-controlled incubators. The AR line was generated by expressing human *AR* gene containing 52 polyglutamine repeats (AR52Q) in *Drosophila* [44]. Eye images were taken with a Leica M205C dissection microscope equipped with a Leica DFC450 camera and were analyzed using the Leica software. The quantification of external eye degenerative phenotype was done using criteria including ommatidial degeneration, brittle, loss of pigmentation, area of degenerated eyes, and disorganization [44].

### Statistical analysis

The data are presented as mean ± SEM. Statistical analysis has been performed using the one-way analysis of variance (one-way ANOVA) to compare 3 or more groups. When one-way ANOVA was significant, we performed a Fisher’s LSD post hoc test. P value < 0.05 was considered statistically significant. Graphical visualization of the statistical significance has been reported as following: *p < 0.05, **p < 0.01, ***p < 0.001, ****p < 0.0001. All the analyses were done with the PRISM (version 5) software. See specific legends for details.

## Results

### AR-A is an androgen receptor isoform abundant in the spinal cord and brainstem of wild type and knock-in mices

The structure of the *AR* gene, the *AR* transcript, and the two AR isoforms are reported in Fig. 1**A-B**, showing that the I-AUG drives the synthesis of the AR-B isoform (ARpolyQ in SBMA patients) and the II-AUG(^Met188^^)^ controls the production of AR-A [27,28]. Since no information is currently available about AR-A expression in adult neuronal and muscular tissues, we compared AR-B and AR-A expression *in vivo* in classical androgen-dependent tissues and in those potentially vulnerable to SBMA. We analyzed the two isoforms in testis, prostate, skeletal muscle (quadriceps), heart, spinal cord (SC), and brainstem (BS) collected at three different ages (1-month-old, 3-month-old and 6-month-old animals) for wild-type (WT) male mice (Fig. 1**C-E**) and at 3-month-old for knock-in (KI) male mice. For these analyses, four mice were utilized per experimental group. Data are presented as mean ± SEM and were analyzed via one-way ANOVA with Fisher’s LSD post-hoc test. By WB analysis in denaturing conditions, AR-B was detected at about 110–114 kDa, whereas the predicted AR-A molecular weight is approximately 87 kDa. To confirm that the band detected at approximately 87 kDa corresponds to the AR-A isoform, we performed a wb analysis comparing the size of the endogenous AR-A with a sample where AR-A was exogenously overexpressed. As shown in Fig. S1**A**, the band detected in the endogenous tissue samples corresponded precisely in molecular weight to the overexpressed AR-A, thus validating our identification of the isoform.

**Fig. 1:**
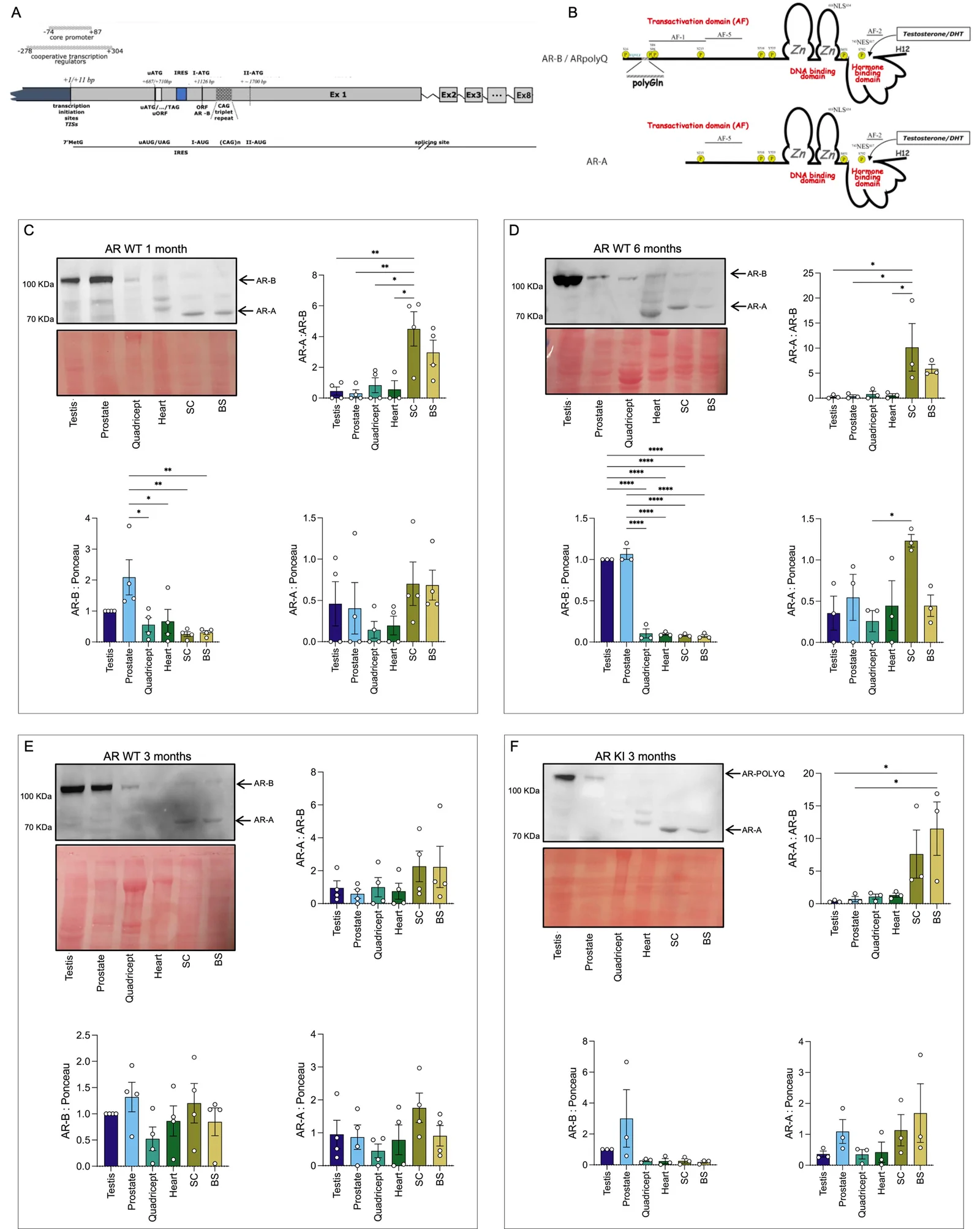
**Differential tissue expression of the AR-B and AR-A isoforms in mouse models** (A) Graphical representation of AR gene, mRNA, and proteins. Localization of the first and second AUG sites and the CAG triplet repeats were reported in the AR mRNA representation. The different initial methionine was reported for both AR-B and AR-A isoforms. (B) Graphical representation of AR-B and AR-A domains and phosphorylation sites. (C-F) Representative WB of the differential expression of AR-B and AR-A in Testis, Prostate, Quadriceps, Heart, SC, and BS of 1-month, 3-months and 6-months wt mice and 3-months KI mice. Graphs represent the quantification of AR-B (lower left graph), AR-A (lower right graph), and AR-A : AR-B ratio (upper right graph) expression in different tissues. Ponceau was used as a loading control; n = 4 ± SEM (*p < 0.05, **p < 0.01, ****p < 0.0001 one-way ANOVA followed by Fisher’s LSD test). (C) Representative WB and quantification of the differential expression of AR-B and AR-A in 1-month wt mice. (D). Representative WB and quantification of the differential expression of AR-B and AR-A in 6-months wt mice. (E) Representative WB and quantification of the differential expression of AR-B and AR-A in 3-months wt mice. (F) Representative WB and quantification of the differential expression of AR-B and AR-A in 3-months KI mice.

We detected AR-B in prostate, testis, skeletal muscle, BS, and SC, while AR-A was detectable only in the SC and BS. By quantifying the levels of AR-B, AR-A, and the AR-A : AR-B ratio, we demonstrated that AR-B is expressed at high levels in testis and prostate, as expected, and at lower levels in quadriceps, heart, BS, and SC in 1-month-old (Fig. 1**C**) and 6-month-old (Fig. 1**D**) WT mice. On the other hand, AR-A expression is detectable only in SC and BS in WT mice at 1 month and 6 months of age. Interestingly, an increase in the AR-A: AR-B ratio was detected in SC and BS of both 1-month and 6-month WT mice (Fig. 1**C- D**).

We extended our analysis to includeKI mouse model of SBMA, expressing the mutant ARpolyQ, alongside WT mice at 3 months of age (Fig. 1**E-F**). In the WT animals, we observed a slight, non-statistically significant increase in the AR-A: AR-B ratio (Fig. 1**E**) in both SC and BS at 3 months compared to 1- and 6-month-old animals. Additionally, we detected a statistically significant increase in the AR-A: AR-B ratio in the SC and BS of the KI animals (Fig. 1**F**).

These results suggest that in both WT and KI mice, the full-length AR-B isoform is the most abundant in non-neuronal tissues, whereas AR-A seems to be more abundant in SC and BS.

### Androgen receptor isoforms intracellular localization and transcriptional activity

While the molecular and biochemical behavior of both AR-B and ARpolyQ are well characterized, few data are available for the shorter AR-A isoform. We thus initially evaluated the physiological activation of AR-A in response to androgens, as well as any potential alterations associated with the lack of the first 188 aa by analyzing its intracellular localization, androgenic activity, and aggregation propensity either under basal conditions or after DHT treatment. In parallel, we assessed similarities and differences between the various AR species considered using the same experimental paradigms.

Both AR-B and ARpolyQ showed diffused cytoplasmic intracellular localization under basal conditions and, as expected, following DHT treatment, AR-B completely translocated into the nucleus and ARpolyQ formed large aggregates and only partially translocated into the nucleus (Fig. 2**A-B**); this suggests that both normally folded and misfolded fractions are generated after androgen activation of the mutant AR. Interestingly, AR-A also exhibited diffused cytoplasmic localization under basal conditions, comparable to AR-B and ARpolyQ behavior (Fig. 2**A-B**). Since AR is maintained in the cytoplasm through its binding to HSPs, this suggests that the lack of the first 188 aa containing the polyQ and the FQNLF motif involved in HSP70 binding (and in the SRC-3 and N-C interaction) does not influence the generation of the typical conformation of the native AR, including NLS inactivation [18,36]. Importantly, upon DHT treatment, AR-A completely translocated into the nucleus. No AR-A aggregates were found in this condition, confirming that the absence of the polyQ in the N-terminus is required and sufficient to have a normal biochemical behavior like AR-B (Fig. 2**A-B**)*.*

**Fig. 2:**
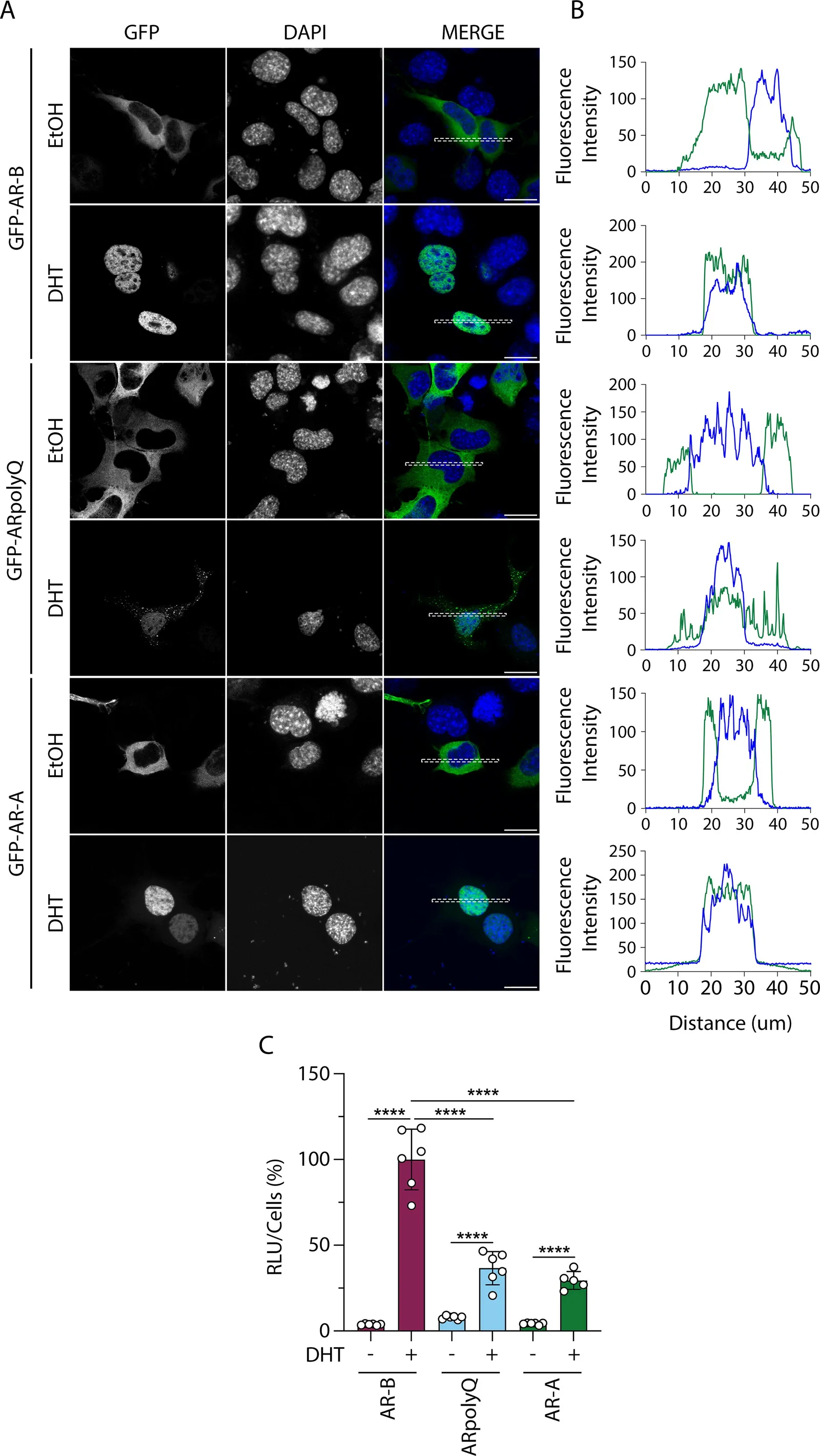
**Intracellular localization of the different androgen receptor isoforms and their transcriptional activity (A)** Fluorescence analysis (63 x magnification) on NSC-34 cells transfected with GFP-AR-B, GFP-ARpolyQ, or GFP-AR-A and treated with DHT (10 nM for 48 h). Nuclei were stained with DAPI (blue). Scale bar: 5μm (B) Relative plot analysis (ImageJ) representing the subcellular distribution of the different AR isoforms. (C) Graph represents the transcriptional induction of a canonical ARE due to the activation of the different AR isoforms upon DHT treatment (10 nM for 48 h); n = 6 ± SEM (****p < 0.0001, one-way ANOVA followed by Fisher’s LSD test).

We next investigated whether AR-A retains the androgenic activity on AR responsive promoters using a plasmid containing a luciferase reporter gene driven by two AREs from the tyrosine amino transferase promoter, followed by the adenovirus E1b TATA box, which is typically utilized to evaluate a classical ARE-mediated response. As reported in Fig. 2**C**, low basal luciferase activity was measured in untreated samples for all the three AR forms tested. Conversely, AR-B activation resulted in a robust increase of luciferase expression upon DHT treatment, as showed by the transcriptional assay. Also in the case of ARpolyQ, we observed transcription induction, but at approximately 40–50 % of AR-B levels. These data are consistent with previous reports, including ours [33,[45], [46], [47], [48]], and are indicative of the LOF already reported for the pathological ARpolyQ isoform, which possibly associates to a repressive function of the polyQ tract on transcription. With regards to AR-A activity, we found that this isoform can activate the ARE-mediated transcription to levels comparable to those observed for the ARpolyQ. This behavior could be related to the partial loss of N-terminal AF-1 domain in the AR-A isoform, which normally acts together with AF-2 to activate gene expression [49].

Altogether, these data demonstrate that AR-A and AR-B have a similar biochemical behavior, as supported by the comparable subcellular localization in both inactive and active states, and that AR-A does not aggregate in all conditions tested. Additionally, AR-A retains an androgenic activity comparable to that measured for the mutant ARpolyQ.

### AR-A biochemical behaviour and preferential degradative pathways

Despite confocal microscopy analysis showed no aggregates in cells expressing AR-A, we sought to test the possibility that this isoform may generate small oligomers not visible with imaging techniques. To this purpose, we checked for the presence of AR high molecular weight (HMW) oligomeric/heteromeric species that can be retained upon filtration through a cellulose acetate membrane with a 0.22 µm pore size, using FRA.

As expected, FRA data showed low levels of AR-B HMW species, both under basal conditions and upon DHT treatment. Also, the ARpolyQ HMW species remained low under basal conditions; conversely, DHT treatment resulted in a robust accumulation of HMW species. This confirms the formation of large amounts of oligomers and aggregated forms of ARpolyQ, indicating that the presence of the elongated polyQ tract alters the solubility of the protein, leading to aggregate formation as already reported [[50], [51], [52]]. When we analyzed AR-A behavior in FRA, we found that, similarly to AR-B, AR-A formed very few HMW species both under basal conditions and upon DHT treatment (Fig. 3**A**). This corroborates the hypothesis that the lack of the NTD containing the polyQ prevents any form of AR-A oligomerization and aggregation, even upon its activation by androgens.

**Fig. 3:**
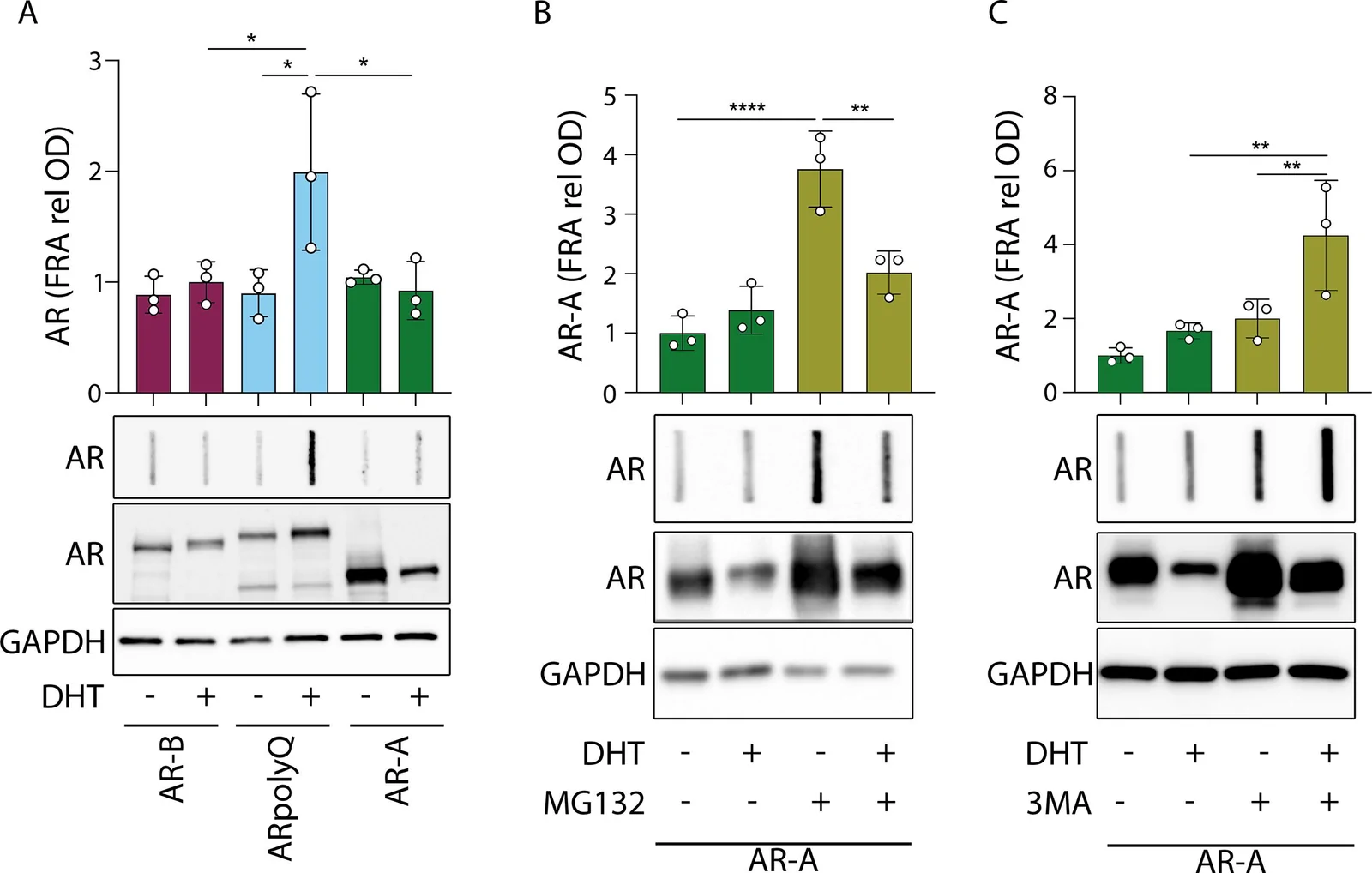
**Aggregation propensity of the AR-A isoform and its preferential degradative pathways** (A-C) NSC-34 cells were transfected with AR-B, ARpolyQ, and AR-A and treated with DHT (10 nM for 48 h). (A) Graph (upper inset) represents the quantification of the AR high-molecular-weight aggregate levels (middle inset), detected with anti-AR antibody (ab52615); n = 3 ± SEM (*p < 0.05, one-way ANOVA followed by Fisher’s LSD test). The correct expression of the different AR isoforms has been evaluated through WB (lower inset). GAPDH was used as a loading control. (B-C) After transfection, cells were treated with MG132 (10 μM for 16 h) or 3MA (10 mM for 16 h). Graph (upper inset) represents the quantification of the AR high-molecular-weight aggregate levels (middle inset), detected with anti-AR antibody (ab52615); n = 3 ± SEM (**p < 0.01, ****p < 0.0001, one-way ANOVA followed by Fisher’s LSD test). The correct expression of the different AR isoforms has been evaluated through WB (lower inset). GAPDH was used as a loading control.

AR activity and behavior could also be altered by its protein turnover. Our data, previously obtained in murine immortalized motoneurons [53,54], demonstrated a preferential proteasomal clearance of AR-B and ARpolyQ inactive forms, since in both cases very high levels of insoluble species were found to accumulate upon proteasomal inhibition. Conversely, upon androgen treatment, the activated ARpolyQ aggregates. Initially, inclusions act as dynamic aggregates (possibly condensates) [40,42,55], which may gradually evolve into insoluble inclusions perturbing intracellular processes [2,55], as well as the autophagy flux, giving rise to a vicious cycle of toxicity [51,52,54,56]. This evidence highlights the difference between the two main degradative pathways involved in ARpolyQ degradation depending on the activation state of the AR. Thus, we focused our attention on evaluating if the lack of the first 188 aa of the NTD in AR-A alters AR selective clearance by the two degradative pathways. While no accumulation was reported for inactive AR-A, proteasome inhibition resulted in its accumulation at very high levels, suggesting that the proteasome might be the preferential degradative pathway for monomeric AR-A. Conversely, the levels of active AR-A were not modified by proteasome inhibition, suggesting that dimeric AR-A might be preferentially degraded through an alternative degradative pathway (Fig. 3**B**). For this reason, we evaluated the effect of autophagy on AR-A degradation. Interestingly, monomeric AR-A levels were not modified by autophagy inhibition, corroborating the hypothesis whereby inactive AR-A is preferentially degraded through the proteasome. On the other hand, active AR-A showed strong accumulation upon autophagy inhibition, suggesting that autophagy is the preferential degradative pathway for the activated form of AR-A (Fig. 3**C**). Interestingly, this difference in the degradative pathways through which AR-A is degraded recapitulates what we previously reported for the other ARs [53]. This suggests that AR-A degradation is not altered by the lack of the first 188 aa of the NTD, including the polyQ tract.

### AR-A is a highly phosphorylated protein

AR-B is highly phosphorylated soon after synthesis. Newly synthetized AR-B can be resolved by SDS-PAGE as a band running around 110 kDa (Fig. 4**A**). Phosphorylation results in the formation of species running around 112 and 114 kDa, including serine 96 that is missing in AR-A [57,58]. We wondered whether AR-A is phosphorylated at similar levels as AR-B. To address this question, we transiently transfected HEK293T cells with plasmids encoding AR-B and AR-A. After lysis, we treated the cell protein extracts with lambda phosphatase (λPP), which indiscriminately removes the phosphate groups from proteins. Due to the fact that phosphorylation occurs rapidly, the 110 kDa species is not very abundant compared to the other two species. Treatment of the cells expressing AR-B with λPP resulted in the loss of the upper isoforms in both untreated and DHT-treated cells, as we have previously shown [57], and in the accumulation of the 110 kDa species. Treatment of AR-A expressing cells with λPP did not modify the electrophoretic mobility shift of AR, at least in part because this isoform that does not have S96.

**Fig. 4:**
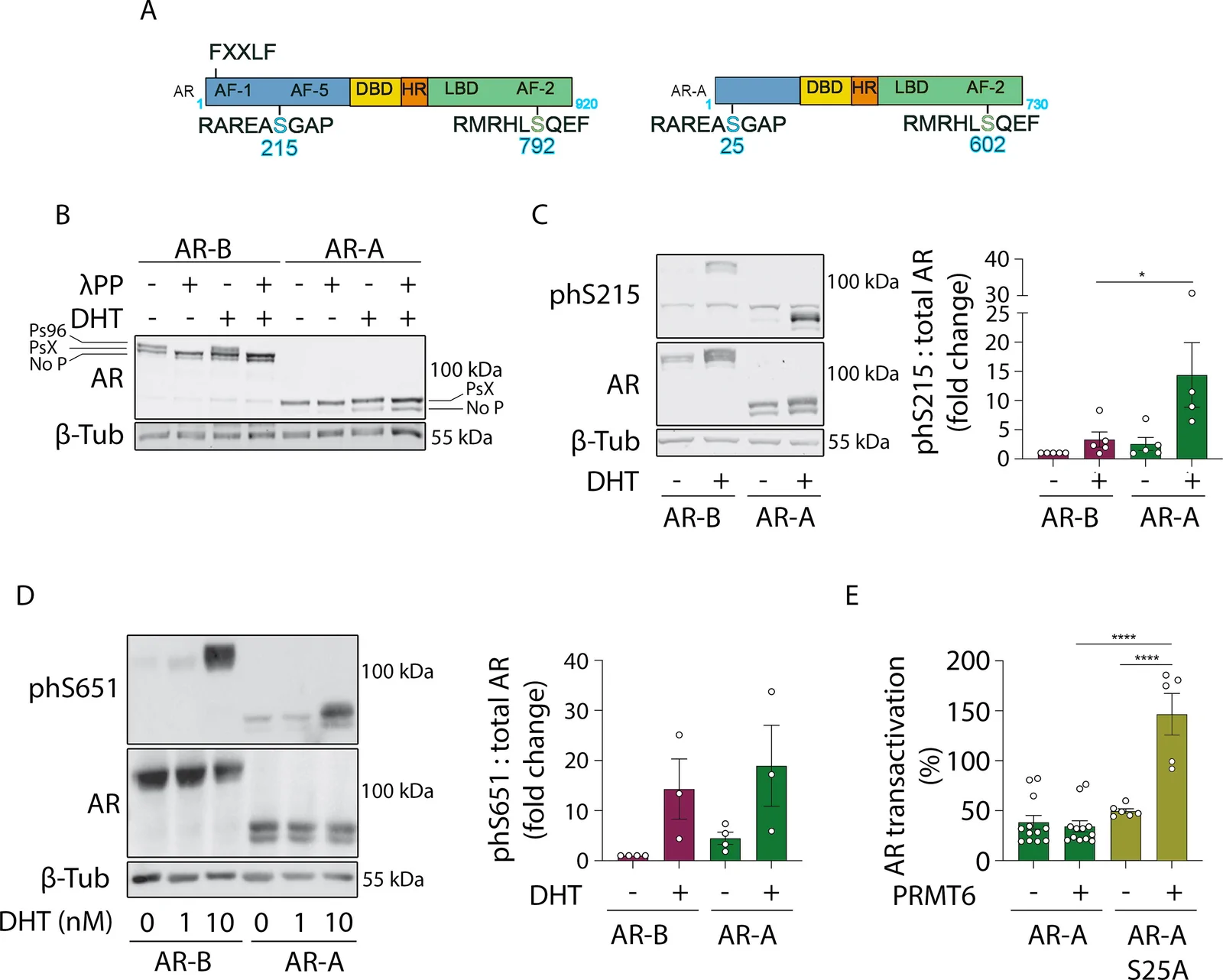
**AR-B and AR-A phosphorylation state and its transcriptional activity mediated by its co-activator PRMT6** (A) Graphical representation of the different phosphorylation sites of AR-B and AR-A. S25 of AR-A corresponds to S215 of AR-B. S602 of AR-A corresponds to S792 of AR-B. (B) Representative WB of HEK cells transfected with AR-B or AR-A and treated with DHT (10 nM for 16 h) and/or lambda phosphatase (λ PP). β-tubulin was used as a loading control. (C) Representative WB and quantification of the phS215: AR ratio in HEK transfected with AR-B and AR-A and treated with DHT (10 nM for 16 h). β-tubulin was used as a loading control. n = 5 ± SEM (*p < 0.05, one-way ANOVA followed by Fisher’s LSD test). (D) Representative WB and quantification of the phS651: AR ratio in NSC-34 transfected with AR-B and AR-A and treated with DHT (1 nM or 10 nM for 16 h). β-tubulin was used as a loading control. (E) Graph represents the transcriptional induction of a canonical ARE in HEK293T cells transfected with AR-A or the phosphor-defective mutant AR-A-S25A and treated with DHT (10 nM for 48 h); n = 6 ± SEM (****p < 0.0001, one-way ANOVA followed by Fisher’s LSD test).

Next, we used specific antibodies that recognize key serine residues when phosphorylated. We have previously shown that AR-B and ARpolyQ are phosphorylated at S215 and S792, which lie in consensus site motifs RxRxxS (where R is arginine and x any amino acid) for the kinase Akt, one located in the NTD, and the other in the LBD of AR [59] **(**Fig. 4**A)**. These sites are of relevance for AR biology and SBMA pathogenesis, as their phosphorylation prevents androgen binding and reduces ARpolyQ toxicity [60]. We thus investigated whether AR-A is phosphorylated at the corresponding serines within the Akt consensus sites, namely S25 and S602 **(**Fig. 4**B)**. We analyzed AR-B and AR-A phosphorylation under basal conditions or upon DHT treatments for 16 h by WB. We confirmed the specificity of the antibody, since it did not recognize AR when S215 was substituted with phospho-defective alanine AR S215A (data not shown). To analyze S792/S602 phosphorylation, we tested all the commercially available antibodies to date, but unfortunately, none of them specifically recognized this phospho-site. Therefore, we focused on S215/S25 phosphorylation (Fig. 4**C)**. As expected S215/S25 phosphorylation levels were low in the absence of ligand and increased significantly by 3.3-fold in response to DHT treatment. Importantly, S25 phosphorylation in AR-A was increased significantly by more than 15-fold in response to DHT treatment. These results suggest that regions included in the first 188 aa of AR negatively regulate AR phosphorylation at this Akt consensus site located in the NTD. Moreover, S25 in AR-A appears to be more exposed at the N terminal of the protein, possibly allowing easier access to Akt and resulting in increased phosphorylation. AR-B is also highly phosphorylated at S651, a ligand-dependent phospho-site located in the hinge region and involved in AR transactivation

[58]. We thus wondered whether AR-A is also phosphorylated at this site, and we found comparable levels of S651/S461 phosphorylation in AR-B and AR-A upon androgen treatment (Fig. 4**D**).

Considering the known role of protein arginine methyltransferase 6 (PRMT6) as a coactivator of both AR-B and ARpolyQ [21], we investigated whether PRMT6 also acts as AR-A coactivator and whether phosphorylation at S25 may modulate this activity. We performed a luciferase assay in HEK293T cells expressing either AR-A or AR-A S25A (in which S25 was substituted with alanine, thus generating a phospho-defective variant) in absence or presence of PRMT6. In presence of androgens, PRMT6 overexpression had no effect on the transactivation properties of AR-A (Fig. 4**E**) At the same time it was able to robustly enhance the transactivation of AR-A in which the S25 was mutated with the phospho-defective AR-A S25A, strongly indicating that phosphorylation at this site prevents the modulation exerted by PRMT6 on AR-A functions. This result is consistent with the increased phosphorylation at S25 of AR-A (Fig. 4**C**), because AR arginine methylation by PRMT6 and phosphorylation by Akt are mutually exclusive [59,61].

### Characterization of androgen receptor heterodimerization

Since AR-A does not aggregate but can potentially heterodimerize with AR-B or ARpolyQ, we evaluated its possible effect as a modulator of ARpolyQ aggregation. In fact, if therapeutic strategies are designed to redirect translation from I-AUG (ARpolyQ) to II-AUG (AR-A), a mixed population of the two isoforms is expected. Thus, we investigated how the co-expression of ARpolyQ and AR-A influences ARpolyQ aggregation and its transcriptional activity. First, we evaluated the effects of different ARpolyQ:AR-A ratios (3:1, 2:2, 1:3) on micro-aggregates and macro-oligomeric species through FRA. The level of aggregate formation in cells expressing both AR isoforms was compared with their relative control represented by the different ARpolyQ : pCDNA3 ratios and an antibody against the N-terminus (aa 1–100) was used to specifically detect ARpolyQ aggregates **(**Fig. 5**A)**. Notably, in these titration experiments, total AR-A protein levels remained relatively stable across the different ratios despite increasing amounts of transfected AR-A plasmid (Fig. 5**A**). This is in line with the experimental setup since the AR-A and ARpolyQ can dimerize and ARpolyQ sequester AR-A from degradation. However, with the increased levels of AR-A also ARpolyQ remains more soluble and the steady-state levels in these condition (which is detected in WB) results in similar AR-A levels among the samples. In fact, as the AR-A plasmid concentration increases, there is a concomitant reduction in the co-transfected ARpolyQ plasmid; thus, the increase in exogenous AR-A expression is counterbalanced by a decrease in the AR-A fraction

**Fig. 5:**
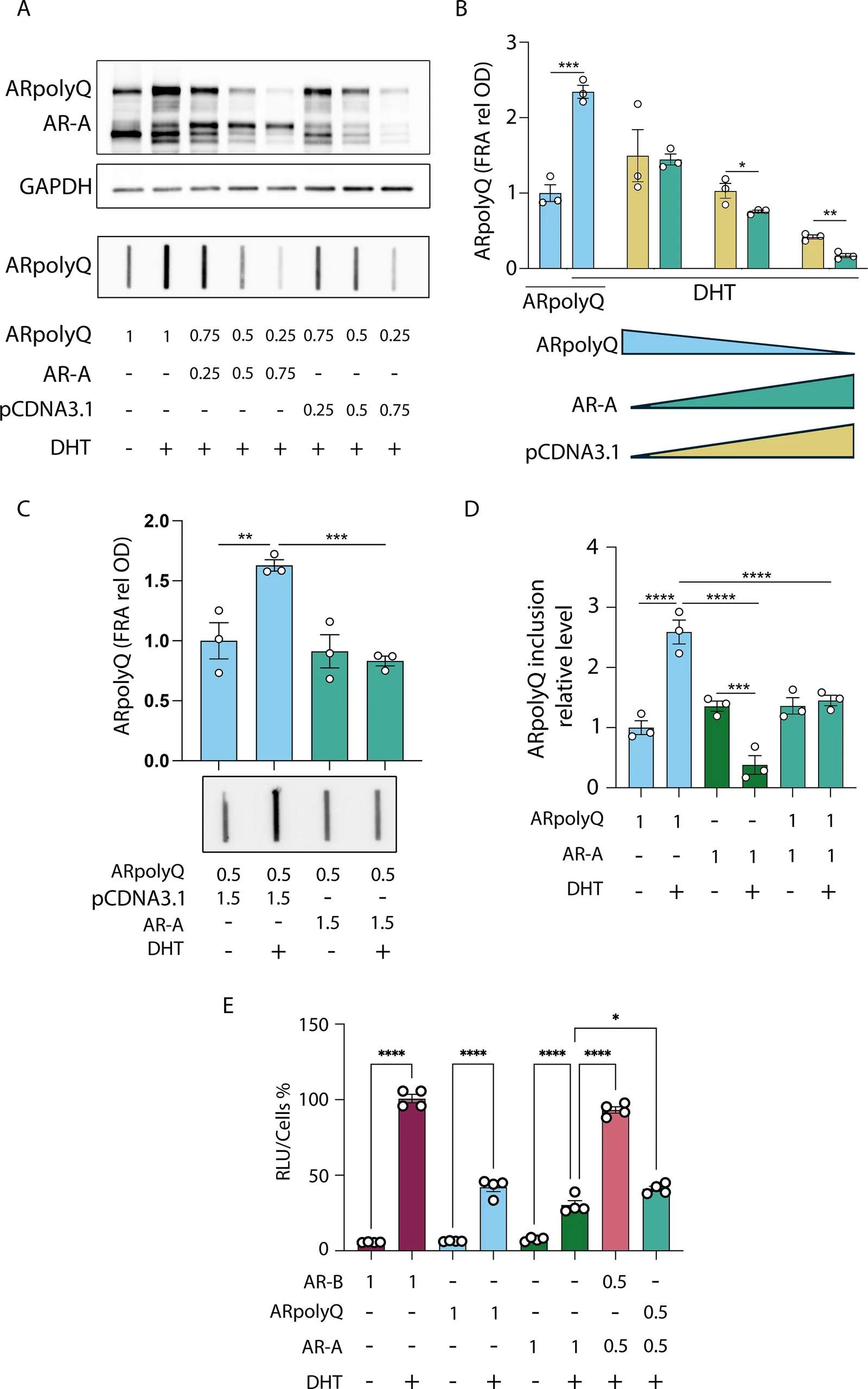
**Evaluation of the androgen receptor heterodimer behavior** (A-B) NSC-34 cells were transfected with different ARpolyQ: AR-A ratios and treated with DHT (10 nM for 48 h). (A) FRA (lower inset) of each ARpolyQ : AR-A ratios visualized with anti-AR antibody (ab133273). The correct expression of the different AR isoforms has been evaluated through WB (upper inset). GAPDH was used as a loading control. (B) Graph represents the quantification of the ARpolyQ high-molecular-weight aggregate levels detected in A; n = 3 ± SEM (*p < 0.05, **p < 0.01, ***p < 0.001, one-way ANOVA followed by Fisher’s LSD test). (C) NSC-34 cells where transfected with 1 : 3 ARpolyQ : AR-A ratio and treated with DHT (10 nM for 48 h). FRA(lower inset) visualized with anti-AR antibody (ab133273). Graph (upper inset) rapresents the quantification of ARpolyQ high-molecular weight aggregate levels; n= 3 (**p < 0.01, *** p < 0.001, one-way ANOVA followed by Fisher's LSD test) (D) Graph represents the quantification of total ARpolyQ aggregates detected through FloIT. Cells were transfected with a 1:1 ARpolyQ: AR-A ratio; n = 3 ± SEM (****p < 0.0001, one-way ANOVA followed by Fisher’s LSD test). (E) Graph represents the transcriptional induction of a canonical ARE due to the activation of the different AR homodimers and heterodimers upon DHT treatment (10 nM for 48 h); n = 4 ± SEM (*p < 0.05, ****p < 0.0001, one-way ANOVA followed by Fisher’s LSD test).

Upon DHT treatment, reduction in ARpolyQ protein levels was coupled with diminished aggregate formation **(**Fig. 5**A-B)**. Interestingly, the co-expression of ARpolyQ and AR-A reduced to a greater extent ARpolyQ aggregates compared to controls **(**Fig. 5**A-B).** These data suggest that AR-A prevent ARpolyQ insoluble oligomeric specie formation. By quantifying the ARpolyQ:AR-A ratio-dependent macro-oligomeric species formation **(**Fig. 5**B),** we found that a 25 % reduction of ARpolyQ was sufficient to significantly reduce ARpolyQ macro-oligomeric species. However, the substitution of this 25 % with AR-A was not sufficient to enhance this reduction. In contrast, a 50 % reduction of ARpolyQ associated with a 50 % increase in AR-A expression resulted to be sufficient to detect the antiaggregant/pro-solubilizing effect of AR-A on ARpolyQ. However, in the 3:1 ratio (ARpolyQ : AR-A), the transfection of 0.25 μg of ARpolyQ resulted in low basal aggregate levels, which potentially limited our ability to resolve the pro-solubilizing effect of AR-A at this specific concentration. Therefore, we further investigated this activity by increasing the starting amount of ARpolyQ to 0.5 μg and co-expressing it with 1.5 μg of AR-A. As shown in Fig. 5C, the co-expression of ARpolyQ and AR-A in this 1:3 ratio led to a robust reduction in aggregate formation, providing further validation of the pro-solubilizing effect of AR-A. These data suggest that an increase of AR-A levels has a potent pro-solubilizing effect on ARpolyQ insoluble species.

We then evaluated AR-A pro-solubilizing effect on ARpolyQ detergent-insoluble and mature aggregates using the flow cytometric analysis of inclusions and trafficking (FloIT) technique. To quantify changes in ARpolyQ aggregates, we co-expressed a GFP-tagged ARpolyQ construct with an untagged AR-A. Upon DHT activation, cells expressing GFP-ARpolyQ were characterized by increased levels of large detergent-insoluble ARpolyQ species, while in cells expressing GFP-AR-A no detergent-insoluble AR-A species could be detected. Interestingly, an increase in the mature aggregates of ARpolyQ could not be detected in cells co-expressing both isoforms **(**Fig. 5**D).**

These data corroborate the hypothesis that AR-A has a pro-solubilizing effect on ARpolyQ aggregates and indicates that AR-A also acts on different aggregate types (micro-aggregates, macro-oligomeric species, and detergent-insoluble mature forms).

As previously mentioned, AR-A retains a partial androgenic activity. Conversely, the shorter AR45 isoform, characterized by a complete depletion of the NTD, shows an inhibitory effect on AR-B transcriptional activity, exerting a dominant negative effect on AR-B [26]. To evaluate if AR-A also has a dominant negative effect on ARpolyQ, we performed a transcriptional activity assay in which the various AR isoforms were co-expressed. The data obtained with the three homodimers of the different AR isoforms showed that ARpolyQ and AR-A are characterized by a 60 % and a 70 % reduction of the transcriptional activity, respectively, if compared with AR-B. Interestingly, when AR-A was co-expressed with both AR-B and ARpolyQ, their original transcriptional activity levels remained unmodified. Indeed, if the transcriptional activity of the AR-B homodimer is set to 100 %, AR-B : AR-A heterodimers retain 93 % of this activity, but ARpolyQ homodimers and ARpolyQ : AR-A heterodimers preserve around 40 % of this activity **(**Fig. 5**D).** These data suggest that, despite the lack of the 188 aa of the NTD, AR-A has no dominant negative effect on AR-B or ARpolyQ.

### AR-A exerts a pro-solubilizing effect on ARpolyQ aggregates

We next sought to better characterize the pro-solubilizing effect of AR-A on ARpolyQ aggregates, as it could have therapeutic benefit in SBMA.

GFP-tagged AR-B and ARpolyQ in combination with RFP-tagged AR-A were used upon co-expression to evaluate heterodimer intracellular localization, nuclear translocation, and aggregate formation.

GFP-AR-B and GFP-ARpolyQ exhibit a comparable behavior to the one previously reported (see Fig. 2**A**). Importantly, RFP-AR-A exhibits a superimposable behavior to that of the GFP-AR-A, suggesting that different fluorescent tags do not alter AR-A localization and translocation **(**Fig. 6**A)**. Interestingly, cells co-expressing both GFP-ARpolyQ and RFP-AR-A were characterized by a diffused cytoplasmic localization of AR and, after DHT activation, by a complete nuclear translocation associated with few aggregates formation **(**Fig. 6**A)**. The Fiji plugin Aggrecount was used to evaluate the number of aggregates per cell, thus characterizing the different conditions. Notably, the mean number of aggregates present in cells expressing GFP-ARpolyQ was 179.1 ± 75.5, while in cells expressing both GFP-ARpolyQ and RFP-AR-A it was 23.6 ± 14, which corresponds to a 90 % aggregates reduction **(**Fig. 6**C)**. This is also associated with a 35 % reduction in the number of cells with ARpolyQ aggregates. Indeed, 60 % of the total cells expressing GFP-ARpolyQ presented aggregates in comparison to 25 % of the total cells expressing both GFP-ARpolyQ and RFP-AR-A **(**Fig. 6**B)**.

**Fig. 6:**
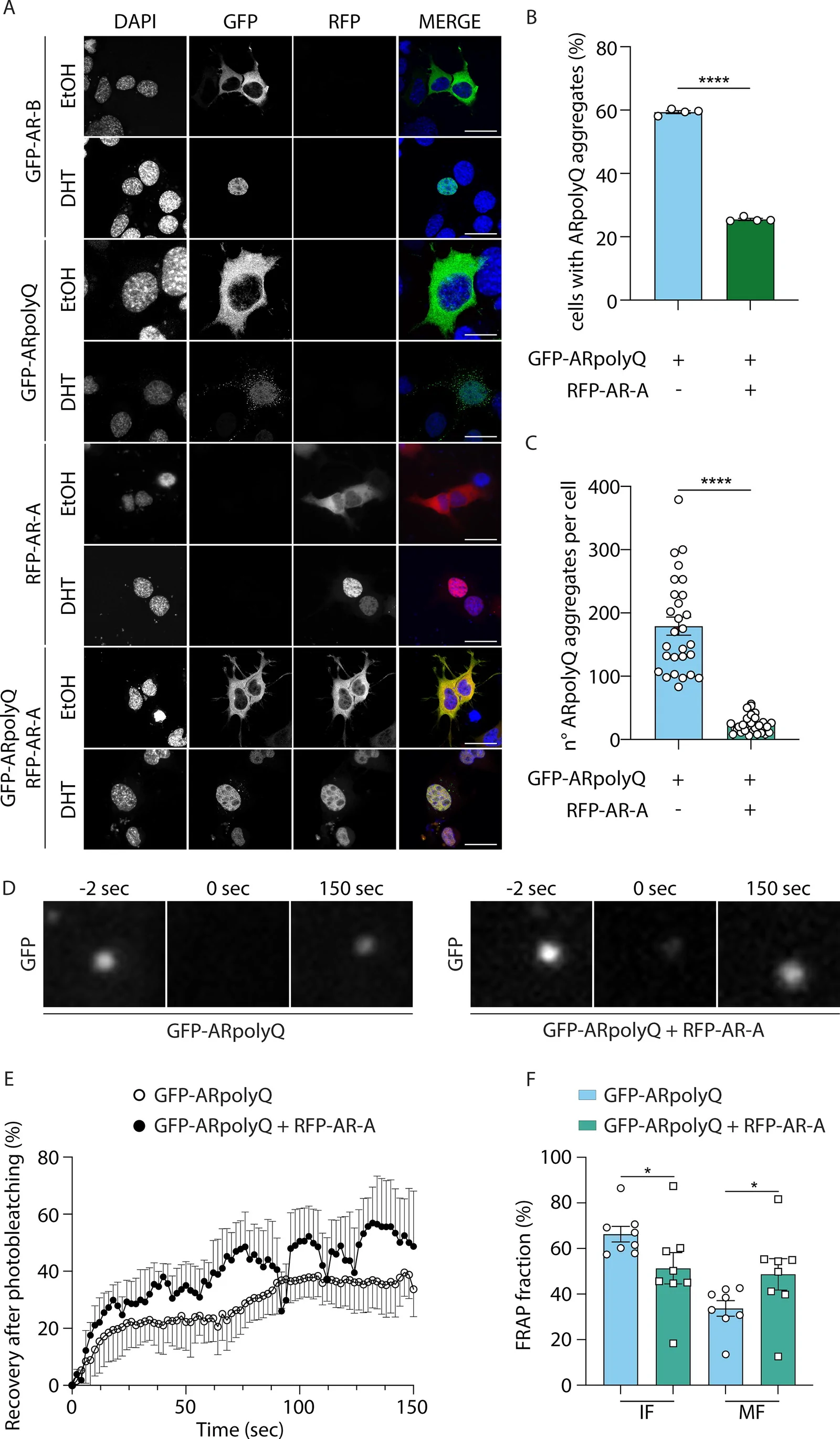
**Evaluation of the pro-solubilizing effect of AR-A on ARpolyQ aggregates** (A) Fluorescence analysis (63x magnification) on NSC-34 cells transfected with GFP-AR-B, GFP-ARpolyQ, RFP-AR-A or co-transfected with GFP-ARpolyQ and RFP-AR-A and treated with DHT (10 nM for 48 h). Nuclei were stained with DAPI (blue). Scale bar: 5μm (B) Graph represents the percentage of cells with ARpolyQ aggregates over 4 independent biological replicates in cells treated with DHT (10 nM for 48 h); n = 4 ± SEM (****p < 0.0001, one-way ANOVA followed by Fisher’s LSD test) (C) Graph represents the quantification of the number of ARpolyQ aggregates per cells over 3 independent biological replicates; n = 3 ± SEM (****p < 0.0001, one-way ANOVA followed by Fisher’s LSD test) (D) Representative image of the recovery after photobleaching of ARpolyQ aggregates in NSC-34 cells transfected with GFP-ARpolyQ or co-transfected with GFP-AR-A and treated with DHT (10 nM for 48 h). Images represent the pre-bleaching (−2 sec), post-bleaching (0 sec), and recovery (150 sec) conditions. (E) Graph represents the percentage of recovery after photobleaching recorded every 2 s. (F) Graph represents the percentage of the immobile and mobile fractions (FRAP fractions) in NSC-34 cells transfected with GFP-ARpolyQ or co-transfected with GFP-AR-A after 150 sec of recovery after photobleaching; n = 8 ± SEM (*p < 0.05, one-way ANOVA followed by Fisher’s LSD test).

We then investigated the possible mechanism involved in the AR-A pro-solubilizing activity on ARpolyQ through the evaluation of aggregate dynamic. Thus, we analyzed the fluorescence recovery after photobleaching (FRAP) and the mobile (MF) / immobile (IM) fraction percentage of the ARpolyQ aggregates in presence or absence of AR-A **(**Fig. 6**D-F).** Of note, ARpolyQ aggregates in cells co-expressing ARpolyQ and AR-A were characterized by a much faster fluorescence recovery after photobleaching than those formed by ARpolyQ alone. Indeed, a mean recovery of 49 % was observed in ARpolyQ : AR-A aggregates, while the mean recovery was only 34 % in cells expressing ARpolyQ alone **(**Fig. 6**E)**. Moreover, in cells expressing both isoforms, aggregates are characterized by a 15 % decrease of IF and a 15 % increase of MF **(**Fig. 6**F).** Therefore, the pro-solubilizing effect of AR-A could be related to an increased dynamicity of ARpolyQ aggregates, suggesting that AR-A retains the few ARpolyQ aggregates in their condensate form, preventing them from evolving into insoluble aggregates, thus permitting a better and more efficient removal by the major intracellular degradative systems. However, it remains to be defined if this altered dynamicity reflects in a reduced aggregate formation, increased degradation, or averted aggregate maturation.

### AR-A suppresses neurodegeneration *in vivo*

*In vitro* analyses reported in this article highlight how AR-A does not undergo aggregation due to the partial NTD depletion. However, since AR-A is translated from the same mRNA of the wildtype and mutant AR as an alternative translation product and accounts for only 20 % of total AR, no *in vivo* model expressing only AR-A was available. For this reason, there was no information regarding the effect of the expression of AR-A alone in an animal model. We have previously generated and characterized a *Drosophila* model of SBMA by expressing exogenous ARpolyQ in the eye [44,61]. Here, we generated flies expressing AR with 22 glutamines (AR-B) and AR-A and analyzed their phenotype together with flies expressing AR lacking the polyglutamine tract (ARQ0) or AR with 52 glutamines (ARpolyQ) [21]. As negative control, we used flies expressing the GFP. For these analyses, 27 flies were utilized per experimental group. Data are presented as mean ± SEM and were analyzed via one-way ANOVA with Fisher’s LSD post-hoc test. Ectopic expression of ARQ0 does not cause toxicity in flies fed with DHT, while ARpolyQ expression resulted in a severe phenotype with degeneration of the posterior side of the eyes [21] and as expected, the expression of AR-B caused only mild phenotypes **(**Fig. 7**A).** Interestingly, the expression of AR-A did not cause eye degeneration, supporting the initial *in vivo* hypothesis whereby AR-A is not able to aggregate and should not exert *in vivo* toxicity **(**Fig. 7**A)**.

**Fig. 7:**
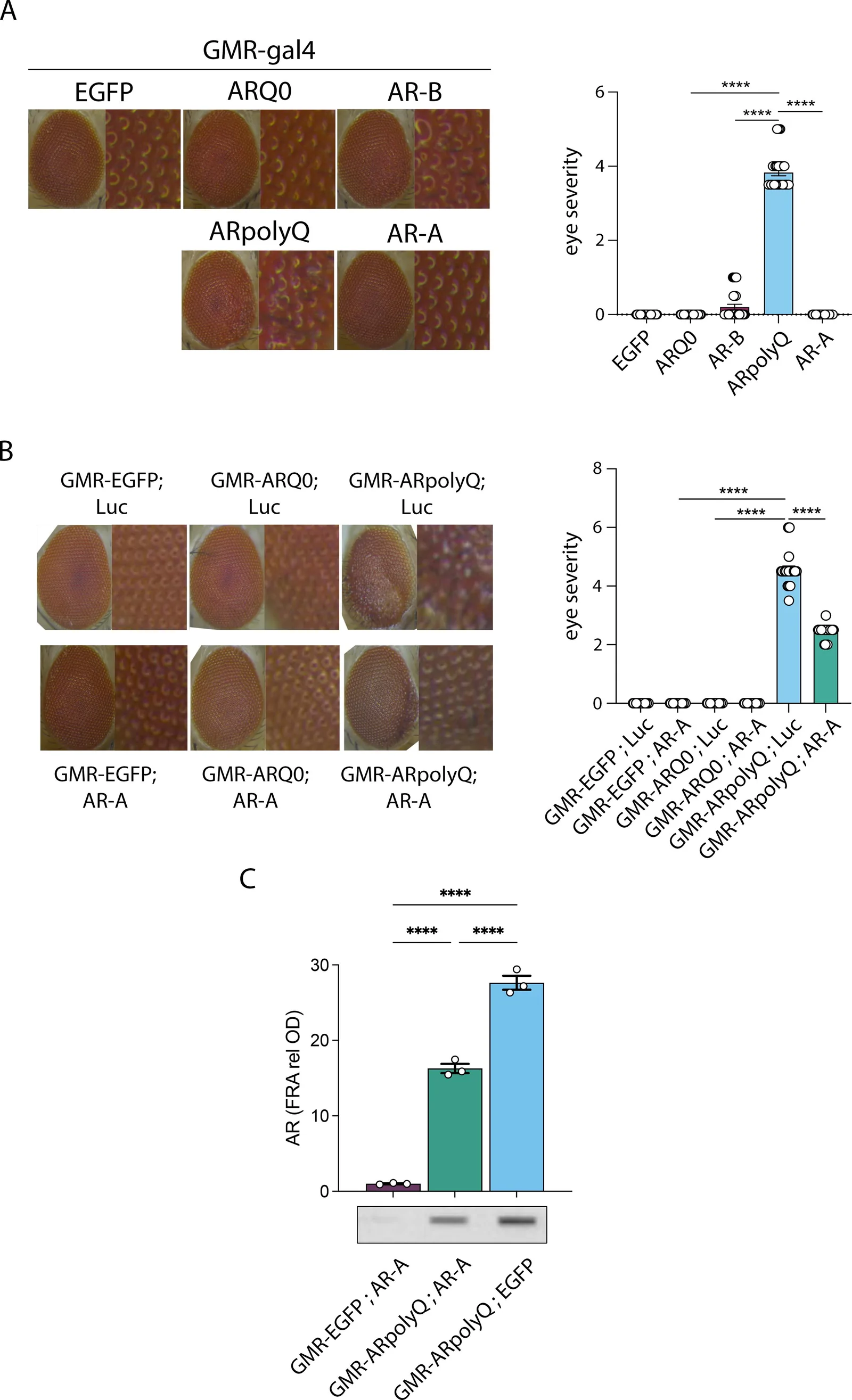
**Evaluation of the pro-solubilizing effect of AR-A on ARpolyQ aggregates *in vivo*** (A) Light microscopy representative images of flies expressing EGFP, ARQ0, AR-B, ARpolyQ, and AR-A (upper inset) and quantification of the eye severity (lower inset); n = 27 ± SEM (****p < 0.0001, one-way ANOVA followed by Fisher’s LSD test). (B) Light microscopy representative images of flies expressing EGFP, ARQ0, ARpolyQ, and AR-A or co-expressing ARpolyQ with ARQ0 or AR-A (upper inset) and quantification of the eye severity (lower inset); n = 20 ± SEM (****p < 0.0001, one-way ANOVA followed by Fisher’s LSD test). (C) Graph represents the quantification of the AR high-molecular-weight aggregate levels detected in *Drosophila* eyes expressing AR-A, ARpolyQ or the combination of the two isoforms; n = 3 ± SEM (****p < 0.0001, one-way ANOVA followed by Fisher’s LSD test).

Next, we tested whether AR-A suppresses the toxicity of ARpolyQ *in vivo* **(**Fig. 7**B).** Thus, we combined the expression of the two transgenes in the eye and measured disease severity in flies fed with DHT. For these analyses, 20 flies were utilized per experimental group. Data are presented as mean ± SEM and were analyzed via one-way ANOVA with Fisher’s LSD post-hoc test. Interestingly, the expression of AR-A led to a 50 % reduction of ARpolyQ-induced neurodegeneration, while no effects were detected when AR-A was co-expressed with ARQ0. To further investigate the mechanism behind the observed suppression of ARpolyQ-induced neurodegeneration by AR-A, we performed a FRA on protein extracts from the *Drosophila* eyes. As shown in Fig. 7C, the co-expression of AR-A with ARpolyQ led to a significant reduction in the amount of high-molecular-weight species compared to eyes expressing ARpolyQ alone. This result provides biochemical evidence that AR-A modifies the aggregation of mutant AR *in vivo*, offering a mechanistic explanation for the reduction in eye degeneration observed under the same conditions (Fig. 7B). Collectively, these findings provide a proof-of-concept that AR-A can modify the neurotoxicity of mutant AR *in vivo* by interfering with its aggregation.

## Discussion

Currently, there are no approved therapies to prevent or halt SBMA progression; patients rely on limited palliative treatments that manage symptoms but fail to address the underlying disease. Most of the proposed therapeutic approaches emerging from preclinical studies are because ARpolyQ toxicity is triggered by androgens and affects only males [40,42,62,63]. Therefore, many approaches rely on the blockage of the gonadal androgen production (e.g., GnRH analogues), the inhibition of AR activation (e.g., antiandrogens), the downregulation of AR synthesis (e.g., antisense oligonucleotides, ASOs), as well as the modulation of degradative pathways to enhance AR clearance [8].

For example, the GnRH analogue Leuprorelin, by inhibiting testosterone synthesis, showed positive effects on MNs degeneration; however, patients show very low compliance to this drug because of depressive symptoms [64]. Casodex (bicalutamide), an FDA-approved drug, has been reported to reduce ARpolyQ levels and aggregates in cellular and murine models through the inhibition of AR nuclear translocation and AR-mediated transcription. Notably, bicalutamide increased the percentage of SBMA mice surviving after one year of treatment and improved both the latency to fall on the rotarod test and the grip strength [65,66]. AJ201 (ASC-JM17) is an activator of central pathways controlling protein folding through the Nrf1/Nrf2 pathway, and it has been reported to reduce mutated AR toxicity [67]. NIDO-361 is a small molecule that binds to AR and acts through a correction of the transcriptional dysregulation to restore healthy cell functions (ClinicalTrials.gov ID: NCT04944940). Moreover, trehalose, a TFEB-mediated autophagy activator, extends the survival and improves the motor performance of KI-ARQ113 mice [65].

These potential therapeutic approaches are often the same as those approved and utilized for prostate cancer and other androgen-dependent pathological conditions, which are based on androgen deprivation therapy (ADT). However, for these conditions they are utilized for a limited therapeutic time window, while in SBMA they should be likely maintained throughout life. Unfortunately, all these current therapeutic approaches which primarily focus on androgen reduction and AR modulation lead to androgen insensitivity and side effects ranging from severe alterations at the endocrine, cardiovascular, and metabolic levels [[68], [69], [70]] to those exerted on muscle trophism [71,72], or imply behavioral changes associated with AR functions in the brain [73]. Therefore, to prevent these adverse effects, it is essential to find a new treatment strategy that operates on ARpolyQ toxicity reduction without lowering AR activity.

In this study, we investigated whether AR-A may preserve androgenic functions, preventing the toxic properties of the mutant ARpolyQ in preclinical models of SBMA. Thus, we analyzed AR-A behavior and activity in counteracting SBMA pathology, with the goal of evaluating whether the expression of AR-A is potentially useful for a novel therapeutic approach in SBMA. Indeed, we demonstrated that, the lack of the 188 aa in the NTD containing the polyQ tract, does not have a detrimental effect on AR behavior, such as misfolding and aggregation. Simultaneously, the presence of most of the NTD and the entire LBD and DBD should preserve most of the AR androgenic activity. While these findings provide a promising proof-of-concept for the therapeutic potential of AR-A in SBMA, our findings are based on preclinical models and lack validation in human-derived samples. Human iPSC-derived MNs or skeletal muscle cells would provide a more physiologically relevant environment to confirm the behavior of AR-A within a human genetic background. Furthermore, moving toward a clinical application requires the development of efficient methods to induce or deliver this isoform. Potential strategies include the use of small molecules, antisense oligonucleotides (ASOs) or SINEUPs designed to specifically trigger the alternative translation of endogenous AR-A. Additionally, the delivery of AR-A through exogenous viral vectors represents a powerful complementary strategy. Investigating these approaches in iPSC-derived MNs and muscles will be a crucial step in determining the feasibility of AR-A as a potential therapeutic approach for SBMA.

Prior studies elucidated how different AR isoforms are physiologically expressed in different human tissues [26]; specifically, AR-A represents 20 % or less of the total AR in various tissues [28]. We describe here that the AR-A isoform is predominantly expressed in the SC and BS, suggesting its possible involvement in mediating AR functions in those structures, even if the actions of the physiological (AR-B) and pathological (ARpolyQ) isoforms of the AR are still poorly understood in these tissues. Nevertheless, AR-A preserves the typical diffuse cytoplasmic localization under basal condition and it can translocate to the nucleus when activated by androgens. Thus, the loss of the first 188 aa in AR-A should not modify the general interaction with chaperones, even if some specific interaction motifs have been described in this region for AR-B [36].

The fact that AR-A completely translocates into the nucleus upon testosterone activation also suggests that it may lead to transcriptional induction of androgen-responsive genes, similarly to AR-B. Conversely, ARpolyQ is characterized by reduced androgenic activity [19,33]. Interestingly, the induction of the AR-responsive transcriptional reporter obtained upon androgenic activation of AR-A is comparable to the one obtained with ARpolyQ activation. The limited androgenic activity of AR-A could be related to the partial loss of the N-terminal AF-1 domain, which normally acts together with the AF-2 to induce gene expression.

Of note, the activity and behavior of the AR can be influenced by its turnover. Our previous research [53] showed that the inactive form of ARpolyQ is primarily cleared by the proteasome, while its active form is mainly degraded by autophagy. This degradative pattern is shared with AR-A; indeed, inactive AR-A accumulates when the proteasome is blocked, while active AR-A accumulates significantly when autophagy is inhibited. This suggests that the lack of a small portion of the NTD in AR-A does not significantly alter the overall degradation mechanisms of this AR isoform.

Phosphorylation is a key post-translational modification of AR. AR is phosphorylated at several sites, including the Akt consensus sites, S215/S25 and S792/S602 [59], and the serine-proline (SP) sites, such as S651 [58]. AR-B and AR-A are both phosphorylated at S651/S461 in response to androgen binding, suggesting that this phospho-site is important not only for AR-B function but also for AR-A. We have previously shown that S215/S25 and S792/S602 prevents androgen binding and suppress AR stabilization, nuclear translocation, and transactivation [59,60]. Additonally phosphorylation at these sites is mutually exclusive with arginine methylation by PRMT6 at the consensus sites, RxRxxS [61]. Conversely, loss of phosphorylation at these sites results in overactivation of AR. Of note, AR-A is much more phosphorylated than AR-B at S25 (which corresponds to S215 in AR-B), and this is consistent with the partial reduction of its transcriptional activity compared to AR-B. Notably, PRMT6 does not transactivate AR-A, but it overactivates a phosphorylation-deficient mutant, further supporting that phosphorylation modulates AR-A function. Whether AR-A is phosphorylated and dephosphorylated by the same kinases and phosphatases that target AR will be the matter of further investigation. However, since S25 is more exposed at the N-terminus of AR-A, this site could be more accessible to kinases, leading to increased phosphorylation.

Since AR-A is characterized by reduced androgenic activity and lack of aggregate formation, we hypothesized that its overexpression could be beneficial for counteracting SBMA pathology. Additionally, AR-A overexpression would result in AR heterodimer formation. In this condition, AR-A exhibits a pro-solubilizing effect on ARpolyQ aggregates, with the most pronounced effect observed when ARpolyQ and AR-A are expressed at equal levels. This suggests that even a 50 % induction of endogenous AR-A, or overexpression through methods like viral delivery, could effectively reduce ARpolyQ aggregation. Of note, this pro-solubilizing effect appears to target various ARpolyQ aggregate types, including macro-oligomers and detergent-insoluble forms. Moreover, AR-A overexpression is associated not only with a 90 % reduction in ARpolyQ aggregates per cell but also with a 35 % decrease in the number of cells exhibiting ARpolyQ aggregates.

Notably, it has been reported that another shorter isoform of the AR (AR45) inhibits the transcriptional activity of the AR-B [26]. However, our data show that AR-A does not inhibit ARpolyQ transcriptional activity but instead resulted in a modest 10 % increase. This enhancement could be attributed to the increased nuclear translocation of AR-A : ARpolyQ heterodimers facilitated by the pro-solubilizing effect of AR-A. We thus investigated the potential mechanism behind this pro-solubilizing effect. Aggregates present in cells co-expressing both AR-A and ARpolyQ are characterized by a 15 % increase in fluorescence recovery after bleaching associated with a 15 % increase in the mobile fraction. These findings suggest that AR-A may enhance ARpolyQ aggregate dynamics.

We subsequently evaluated the effects of AR-A expression in an *in vivo* model. Of note, while eye degeneration (representative of neurodegeneration) is present in ARpolyQ flies, no posterior eye degeneration is induced in flies expressing AR-A, supporting the initial *in vitro* hypothesis that AR-A does not exert toxicity. We finally assessed disease severity in flies expressing both AR-A and ARpolyQ, identifying a 50 % reduction in ARpolyQ-induced neurodegeneration.

In conclusion, our results, primarily derived from cellular and *Drosophila* models, demonstrate that increased expression of AR-A is associated with a significant mitigation of ARpolyQ aggregation and toxicity. This work establishes a crucial molecular proof-of-concept of the protective role of AR-A and suggests a novel, promising therapeutic avenue that may circumvent the undesirable endocrine side effects associated with current androgen-suppressive treatments. However, the protective effect observed across different preclinical models warrants further and more comprehensive *in vivo* validation in appropriate mammalian systems to confirm the long-term safety, efficacy, and translational applicability of AR-A induction

## Conclusion

In the present study, we demonstrated that AR-A, an alternative AR isoform predominantly expressed in the SC and BS, possesses biochemical properties which generally overlap those of the physiological full length AR (AR-B), but lacks those aberrant properties which characterize the mutated ARpolyQ form causing SBMA. Specifically, AR-A does not aggregate and undergoes normal cytoplasmic-to-nuclear translocation upon androgen activation, retaining partial androgen-dependent transcriptional activity. In addition, when AR-A and ARpolyQ are co-expressed in the same cells, AR-A counteracts most of the altered behaviours of the ARpolyQ; for example, it enhances its solubility and clearance preventing its aggregation into insoluble aggregates, possibly by enhanced aggregate dynamicity. We hypothesize that AR-A maintains ARpolyQ in a more dynamic, less aggregation-prone state, thereby facilitating its cellular clearance and preventing the formation of cytotoxic inclusions. Even more interestingly, the heterodimer formed by AR-A : ARpolyQ retains a transcriptional competence comparable to those of the AR-A : AR-A homodimers or ARpolyQ : ARpolyQ, suggesting that if co-expressed together in the same cells AR-A protects against ARpolyQ toxicity in SBMA-affected cells. Our findings thus provide robust data in cell models that AR-A significantly mitigates the ARpolyQ toxicity, that were next confirmed *in vivo* in a *Drosophila melanogaster* SBMA model.

These results highlight how AR-A induction could offer a novel molecular avenue for SBMA, providing a potential alternative to current approaches that often lead to undesirable endocrine side effects. However, its translational potential remains to be validated with further *in vivo* studies to confirm this therapeutic hypothesis. Future research should focus on elucidating the precise molecular mechanisms underlying AR-A pro-solubilizing activity, especially in iPSC patient-derived cells differentiated into MN and muscle cells, to better address AR-A behavior in SBMA-related cells. Furthermore, we will focus on developing strategies to enhance endogenous AR-A expression or deliver it exogenously as a targeted intervention for SBMA with the perspective to evaluate ARpolyQ toxicity mitigation in mice models.

It remains to be determined whether rerouting of translation to favour AR-A production may be useful in other AR-related disorders (i.e. prostate cancer). The fact that the shorter isoform AR-A is able to modulate full-length AR-mediated transcription may be of interest in androgen-responsive prostate cancer (and maybe also in breast cancer, in which AR is expressed in very high levels). Nonetheless, specific studies should be designed to unravel the therapeutic potential of AR-A in these androgen-related human disorders.

## Compliance with ethics requirements

All animal experiments were carried out according to local ethics committees (University of Padova) and the Ministry of Health (Italy) (Approval numbers: 207/2020-PR and 119/2024-PR).

## CRediT authorship contribution statement

**Marta Chierichetti:** Conceptualization, Methodology, Formal analysis, Investigation, Data curation, Visualization, Writing – original draft, Writing – review & editing. **Roberta Andreotti:** Conceptualization, Methodology, Formal analysis, Investigation, Data curation, Visualization, Writing – original draft. **Barbara Tedesco:** Methodology, Resources, Writing – review & editing. **Veronica Ferrari:** Methodology, Resources, Writing – review & editing. **Laura Cornaggia:** Investigation, Data curation, Writing – review & editing. **Paola Pramaggiore:** Writing – review & editing. **Marta Cozzi:** Writing – review & editing. **Ali Mohamed:** Writing – review & editing. **Rocio Magdalena:** Writing – review & editing. **Margherita Piccolella:** Writing – review & editing. **Giulia Boarolo:** Investigation, Writing – review & editing. **Valeria Crippa:** Resources, Funding acquisition, Writing – review & editing. **Paola Rusmini:** Writing – review & editing. **Mariarita Galbiati:** Resources, Funding acquisition, Writing – review & editing. **Carlo Rinaldi:** Conceptualization, Resources, Funding acquisition, Writing – review & editing. **Eric N. Anderson:** Conceptualization, Methodology, Formal analysis, Investigation, Data curation, Visualization, Writing – review & editing. **Udai Bhan Pandey:** Conceptualization, Writing – original draft, Writing – review & editing. **Maria Pennuto:** Conceptualization, Supervision, Funding acquisition, Project administration, Writing – original draft, Writing – review & editing. **Riccardo Cristofani:** Conceptualization, Supervision, Funding acquisition, Methodology, Formal analysis, Investigation, Data curation, Visualization, Writing – original draft, Writing – review & editing. **Angelo Poletti:** Conceptualization, Supervision, Funding acquisition, Project administration, Writing – original draft, Writing – review & editing.

## Declaration of competing interest

The authors declare that they have no known competing financial interests or personal relationships that could have appeared to influence the work reported in this paper.
