# Supplementary material for: The alternative androgen receptor isoform A mitigates toxicity of polyglutamine-elongated mutant androgen receptor in spinal and bulbar muscular atrophy
Source: J Adv Res. 2026 Jan 19;88:959–76. doi: 10.1016/j.jare.2026.01.051 (PMC13669589; doi:10.1016/j.jare.2026.01.051)
Supplement: Supplementary file 1 [file mmc1.docx]

**Figure S1**
